# A Comparative Study of the Diagnostic Value of Contrast-Enhanced Breast MR Imaging and Mammography on Patients with BI-RADS 3–5 Microcalcifications

**DOI:** 10.1371/journal.pone.0111217

**Published:** 2014-11-03

**Authors:** Erni Li, Jing Li, Ying Song, Mei Xue, Chunwu Zhou

**Affiliations:** Department of Diagnostic Radiology, Cancer Hospital, Chinese Academy of Medical Sciences, Beijing, China; University Medical Centre Utrecht, Netherlands

## Abstract

**Objective:**

To retrospectively investigate the diagnostic value of breast MRI in patients with BI-RADS 3–5 microcalcifications in mammography.

**Methods:**

Eighty-four patients with BI-RADS 3–5 microcalcifications on mammography underwent breast MR exams before surgical biopsy with a hookwire position under mammographic guidance. Two radiologists reviewed each lesion with BI-RADS by consensus. The diagnostic value of mammography and MRI was compared.

**Results:**

Histopathological examination revealed 49 benign lesions and 42 malignant lesions. In the assessments of mammography, 21 lesions (23.1%) were assigned to category 3, 51 lesions (56.0%) to category 4, and 19 lesions (20.9%) to category 5. The area under the receiver operating characteristic(ROC) curve for mammography and MR assessment was 0.844, and 0.945, respectively (p<0.05). In cases of category 3 microcalcifications, the specificity of mammography and MR was 100%, and 95.2% (p = 1.000), respectively. In cases of category 4 microcalcifications, the specificity, PPV and accuracy of mammography was 0%, 45.1% and 45.1%; whereas those for MR was 82.1% (p<0.05), 80.8% (P = 0.003) and 86.3% (p<0.05). All microcalcifications of category 5 were correctly diagnosed by mammography and MR.

**Conclusions:**

Breast MRI has the potential to significantly improve the diagnosis of category 4 microcalcifications on mammography. Among mammographic category 4 microcalcifications, about 82% of benign lesions can be degraded to BI-RADS 1∼3 by MRI. However for microcalcifications of category 3 and 5, MR exams do not show significant improvement over mammography.

## Introduction

With the spread of screening mammography, more and more microcalcifications are found and needed further assessment. Mammographically detected microcalcifications are a frequent feature of early diagnosed breast tumors, found in approximately 70% of minimal breast cancers, and frequently in ductal carcinoma in situ (DCIS) [1]. However, the specificity of microcalcifications is low, ranging from 10% to 60% [2].

MRI is becoming an invaluable tool in the diagnosis and treatment of breast disease. Although breast MRI has demonstrated variable specificity, the sensitivity for the demonstration of invasive ductal carcinoma has approached 100% [3]. The role of MRI in characterizing breast microcalcifications remains a debated issue. Previous studies performed at 1.5 Tesla (T) in patients with microcalcifications reported sensitivities between 45% and 95%, and specificities between 68% to 100%, respectively [4]–[8]. Currently, breast imaging is slowly moving towards the higher field strength of 3-T. Comparing with 1.5-T breast MRI, 3-T MR provides better image quality and improves lesion assessment [9]–[10]. To our knowledge, only two reports of 3-T breast MRI in patients with suspicious microcalcifications were reported [11], [12], which stated 3-T MRI increases the diagnostic value in patients with microcalcifications.

In this report, we analyzed 84 patients with mammographic microcalcifications who underwent 3-T MR imaging. The purpose of our study was to compare the diagnostic value of MR and mammography, to evaluate whether 3-T MR imaging can help to detect the presence of malignancy in patients with different categories of microcalcifications on mammography.

## Materials and Methods

### Patient enrollment

Our institutional ethics committee (Ethics Committee of Cancer Institute and Hospital, Chinese Academy of Medical Sciences) approved this retrospective study. Our institutional ethics committe specifically approved the absence of informed consent because of the retrospective nature of the study and also because the data were analyzed anonymously.

The inclusion criteria were patients with suspicious microcalcifications classified as BI-RADS category 3–5 on mammograms, associated with or without an opacity. Dynamic contrast-enhanced MRI was performed within 30 days after or before mammography. All patients received surgical biopsy with mammographic guidance. A total of 84 female patients (aged 25–76, mean age 46) were included at our institution between January 2012 and December 2013. Sixteen patients experienced menopause. Fourteen patients had family history of breast cancer. Among 84 patients, 47 women were for screening; five women complained with breast masses, eight with nipple discharge, 15 with known breast carcinoma; two with follow-up after breast carcinoma operation; four with axillary lymphadenopathy, and three with local excision of breast carcinoma in other hospitals.

### Mammography equipment and scan parameters

Bilateral digital mammography was performed (Senographe DS; GE healthcare, USA) and included routine craniocaudal and mediolateral oblique views of the breasts. Magnification view was routinely performed to assess microcalcifications.

### MRI equipment and scan parameters

MRI was performed with the patients in the prone position. The instrument was a 3.0-T commercially available system (Signa Excite HDx; General Electric, USA) with a dedicated phased-array 8-channel bilateral breast coil. Our imaging protocol consisted of axial T2-weighted single-shot fast-spin echo sequences with fat suppression (TR/TE  = 3800/80; matrices  = 384×224; field of view  = 320 mm; section thickness/interslice gap  = 5 mm/0.5 mm) and dynamic contrast-enhanced MRI. Dynamic contrast-enhanced MRI of bilateral breasts was performed using a three-dimensional (3D) fat-suppressed sagittal VIBRANT (volume imaged breast assessment) sequence (TR/TE  = 4.8/1.9; flip angle  = 10°; matrices  = 288×192; field of view  = 240 mm; section thickness/interslice gap  = 3.6 mm/0 mm). Following the unenhanced baseline acquisition, gadodia midehydrate was administered as an intravenous bolus (dose, 0.1 mmol/kg bodyweight) over 10 s from the dorsum of the hand, followed by a saline flush of 20 ml. The serial contrast-enhanced MR acquisitions were initiated 15 s after the beginning of the injection of contrast medium; nine sequential acquisitions were obtained; a sequence lasted about 40 to 50 seconds. Finally, a fat-suppressed axial delayed-phase sequence (VIBRANT: TR/TE  = 4.8/1.9; flip angle  = 10°; matrices  = 384×384; field of view  = 380 mm; section thickness/inter slice gap  = 3.6 mm/0 mm) was acquired.

### Surgical biopsy

Surgical biopsy rather than percutaneous biopsy is routinely performed in our hospital due to multiple factors including the patients' unwillingness and practical factors. After a hookwire was positioned under mammographic guidance, histopathological diagnosis was obtained in all patients after surgical biopsy. All surgical specimens underwent radiographic examination to confirm the excision of microcalcifications. The lesions were then histopathologically classified as benign or malignant.

### Analysis of cases

All mammography were read by consensus of two experienced radiologists using BI-RADS assessment categories without knowing the MRI findings. All MRI examinations were assessed by the same two radiologists awaring the locations of microcalcifications. The two radiologists were aware of the entry criterion and blinded to all of the patients data and to histopathological results.

### Statistical Analysis

Statistical analysis were performed using software SPSS (version18.0).

BI-RADS 3 lesions were considered benign and BI-RADS 4 and 5 were considered malignant. We used the sensitivity, specificity, positive predictive values (PPV), negative predictive values (NPV), and accuracy to describe the diagnostic value of mammography and MR. Discrimination was estimated with the area under the curve (AUC) of the receiver operating characteristic (ROC) curve based on Hanley and McNeil method. Chi-square and Fisher exact tests were performed for statistical significance, with p<0.05 considered significant.

## Results

In total 91 lesions were found with 49 benign and 42 malignant lesions. Among the 49 benign lesions, fibroadenoma was in three lesions, intraductal papilloma in three, normal breast tissue in four, and adenosis in 39. The size of the microcalcifications ranged between 4 and 100 mm (median, 23 mm) on mammography. For the 42 malignant lesions, 16 were pure DCIS (with one grade 1, five grade 2, eight grade 3, and two unknown grade), seven were DCIS with microinvasion, and the rest 19 were invasive ductal carcinoma. The size of the lesions ranged between 5 and 90 mm (median, 33 mm) on mammography and between 5 and 100 mm (median, 37 mm) on MRI.

### Mammographic findings

In the assessment of mammography based on the BI-RADS categorization of these 91 microcalcifications (Table 1), 21 lesions (23.1%) were assigned to category 3, 51 lesions (56.0%) to category 4, and 19 lesions (20.9%) to category 5.

**Table 1:** Mammographic features of 91 microcalcification lesions.

category	morphology	distribution					total
		diffuse	regional	clustered	linear	segmental	
3	punctate	4	4	5	0	0	13
	amorphous	0	3	0	1	0	4
	coarse	0	1	3	0	0	4
4	punctate	0	2	1	0	0	3
	amorphous	0	5	14	3	7	29
	coarse heterogenous	0	0	2	0	3	5
	pleomorphic	0	1	9	3	0	13
	linear or branch-like	0	0	0	1	0	1
5	amorphous	0	0	1	0	2	3
	pleomorphic	0	0	2	0	7	9
	linear or branch-like	1	0	2	1	3	7

### Dynamic contrast-enhanced MR imaging findings

There was no enhancement (Table 2) in 11 lesions, one of which was DCIS. For 80 lesions showing enhancement, there were 11 masses, of which 72.7% (8/11) were malignant, and 69 non-mass-like enhanced lesions, of which 49.3% (34/69) were malignant. Among breast MR imaging distribution, PPV was highest for segmental (100%, 13/13) and ductal (90.9%, 10/11) enhancement. NPV was highest for diffuse or scattered enhancement (100%, 18/18) and no enhancement (91.7%, 11/12).

**Table 2:** Enhanced MRI features of 91 lesions.

MR category	Enhancement distribution on MR									
	no enhancement	focal regional	linear	ductal	segmental	regional	multiple regional	diffuse	mass	focus
1	11	0	0	0	0	0	0	0	0	0
2	0	0	0	0	0	0	0	11	0	1
3	0	1	0	0	0	3	3	7	3	5
4	0	2	3	4	1	0	1	0	1	5
5	0	1	0	7	12	1	1	0	7	0
Total	11	4	3	11	13	4	5	18	11	11

### Comparison of dynamic contrast-enhanced MR imaging and mammography

The correlation of mammography for microcalcifications with MR and histopathology is listed in Table 3.

**Table 3:** Correlation of mammography for microcalcifications with MR and histopathology.

X-ray category	MR category									
	1		2		3		4		5	
	benign	malignant	benign	malignant	benign	malignant	benign	malignant	benign	malignant
3	3	0	4	0	13	0	1	0	0	0
4	7	1	8	0	8	1	5	6	0	15
5	0	0	0	0	0	0	0	5	0	14

The sensitivity, specificity, PPV, NPV and accuracy of mammography was 100%, 42.9%, 60%, 100% and 69.2%, respectively; whereas that for MR was 95.2%, 87.8%, 87%, 95.6% and 91.2% (Table 4). There were significant differences for specificity, PPV and accuracy between mammography and MR (p<0.05).

**Table 4:** The diagnostic value of mammography and MRI for BI-RADS 3–5 microcalcifications.

	sensitivity	specificity	PPV	NPV	accuracy	AUC
X-ray	100%	42.9%	60%	100%	69.2%	0.844
MR	95.2%	87.8%	87%	95.6%	91.2%	0.945
p	0.474	0.000	0.002	1.000	0.000	0.000

**Figure 1:**
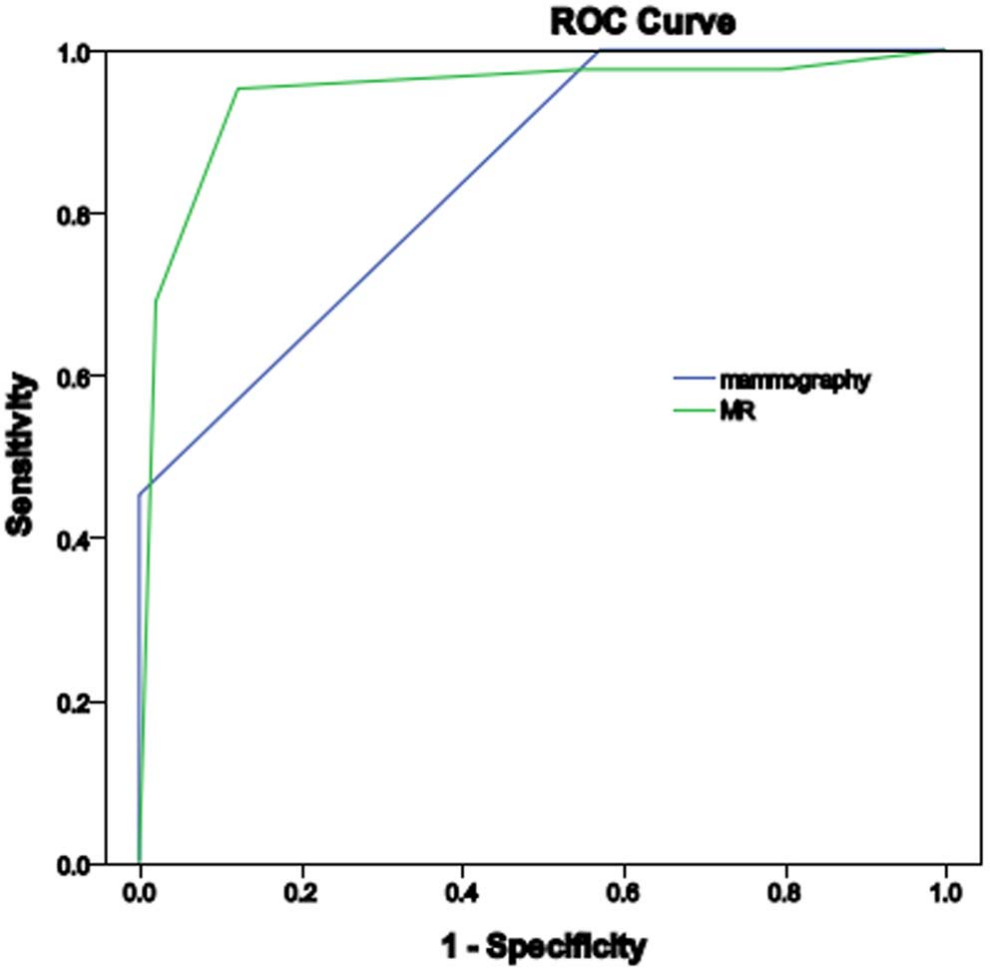
ROC curves for the diagnosis of mammography and MR. The AUC is 0.844 and 0.945, respectively (p<0.05).

The AUC of mammography and breast MRI assessment were 0.844 and 0.945 (Figure 1). The observers performed better on the breast MRI than on the mammography (p<0.05).

21 lesions of category 3 diagnosed by mammography were all proved to be benign by histopathology. On MR, one lesion showing ductal enhancement was upgraded to BI-RADS 4, which were proven to be intraductal papilloma by histology, 13 lesions maintained BI-RADS 3 and the other 7 lesions were degraded to BI-RADS 1 or 2. Therefore, the specificity of mammography and MR was 100% (21/21) and 95.2% (20/21) (p = 1.000). The NPV of mammography and MR was 100% (21/21) and 100% (20/20).

**Figure 2:**
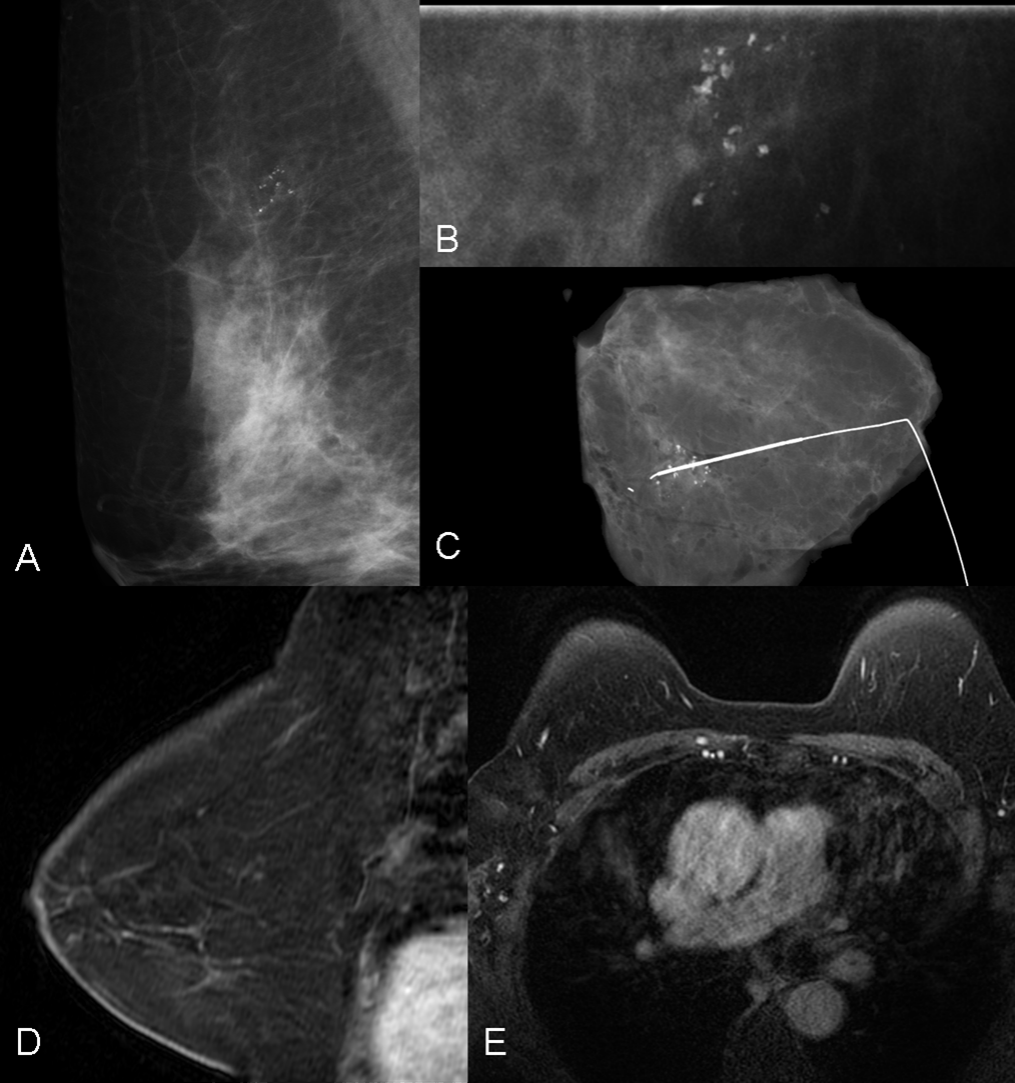
A case of 64-year-old woman who complained with microcalcifications in the right breast on screening mammography. Surgical biopsy suggested breast tissue and microcalcifications within dilated duct. Lateral oblique view (A) and magnified view (B) showed a cluster of fine pleomorphic microcalcifications with the extent of 1.1 cm. Mammographic BI-RADS category 4 was suggested. Surgical specimen showed clusted microcalcifications around the hookwire(C). Absence of contrast uptake in the corresponding location on sagital (D) and axial (E) MRI was observed. BI-RADS-MRI category 1 was diagnosed.

Among 51 lesions of mammographic category 4 (Figure 2), 54.9% (28/51) were benign and 45.1% (23/51) were malignant. The sensitivity, specificity, PPV and accuracy of mammography was 100% (23/23), 0%, 45.1% (23/51), 45.1% (23/51). On MR, 15 lesions were upgraded to BI-RADS 5, which were proved to be malignant; 25 lesions degraded to BI-RADS 1∼3 proven of 23 benign and 2 malignant ones and 11 lesions sustained of BI-RADS 4 verified of 6 malignant ones and 5 benign ones. DCIS were revealed in two false negative cases, one grade 2 and one grade 3. The sensitivity, specificity, PPV and accuracy of MR was 91.3% (21/23), 82.1% (23/28), 80.8% (21/26) and 86.3% (44/51). The difference for specificity, PPV and accuracy between mammography and MRI was significant (*p*<0.05), but not for sensitivity (p = 0.470) (Table 5).

**Table 5:** The diagnostic value of mammography and MRI for BI-RADS 4 microcalcifications.

	sensitivity	specificity	PPV	NPV	accuracy
X-ray	100%	0	45.1%	0	45.1%
MR	91.3%	82.1%	80.8%	92%	86.3%
p	0.470	0.000	0.003	—*	0.000

*—not comparable.

Among 19 lesions of mammographic category 5 which were all proved malignant by histopathology, 5 lesions were degrade to BI-RADS 4 and 14 lesions maintained BI-RADS 5 on MR. The accuracy of mammography and MR was both 100% (19/19).

## Discussion

### Role of mammography for microcalcifications

Mammography is extremely sensitive in detecting microcalcifications even though it can not distinguish malignant from benign lesions and invasive carcinoma from DCIS. The classification of microcalcifications on mammography is complex, mainly based on morphology and distribution characteristics. This classification determines the final assessment of the lesion, histological biopsy or mammographic follow-up. The reported values of specificity for category 3–5 microcalcifications range between 23% and 61.5% and the accuracy, between 42.9% and 69.2% [4]–[8]. Our results were 42.9% and 69.2% respectively. The low specificity of microcalcifications on mammography results in a high number of percutaneous or surgical diagnostic biopsies.

### Role of MR for mammographic microcalcifications

Dynamic contrast-enhanced MRI is an effective diagnostic technique for breast diseases. However its role in evaluating mammographically detected suspicious microcalcifications remains unclear. Previous MR studies have reported variable accuracy of MRI for classification of microcalcifications. Westerhof et al [5] reported a sensitivity of 45%, a specificity of 72% and an accuracy of 56% for dynamic MRI in patients with suspicious microcalcifications. Bazzocchi M et al [6] observed a sensitivity of 87%, a specificity of 68% and an accuracy of 80% for MRI in patients with category 4–5 microcalcifications. Akita A et al [7] reported a sensitivity of 85%, a specificity of 100% and an accuracy of 96% for MRI in patients with category 3–5 microcalcifications. Fiaschetti V et al [8] observed a sensitivity of 88.8% and a specificity of 76.9% for MRI in another study. In our study, MRI had a sensitivity of 95.2%, a specificity of 87.8% and an overall accuracy of 91.2%. This difference may come from the different population of these studies, sequences parameters or inconsistent criteria for MRI evaluation, especially the latter. Bazzocchi M et al [6] used Fischer score as criteria. Akita A et al [7] devised MR classification system and proposed a more detailed definition of non-mass-like enhancement in order to detect DCIS. In study by Fiaschetti V et al [8], the presence or absence of contrast uptake in the breast was the only parameter used to decide if the area of microcalcifications was associated with malignancy. BI-RADS-MR assessment category was used in our study.

In our study, AUC was increased from 0.844 for mammography assessment to 0.945 for MR assessment (p<0.05). Hence, MR significantly improved the diagnostic value of microcalcifications compared with mammography. However, 3.0 T MR showed similar results compared with previous 1.5 T MR studies, which were concordant with the report of Stehouwer BL [11].

#### Category 3 microcalcifications

BI-RADS recommends 6-month follow-up imaging rather than immediate biopsy for category 3 lesions, probably benign lesions. The management of BI-RADS 3 lesions continues to be controversial [13]–[14]. Several studies reported that patients with BI-RADS category 3 microcalcifications should undergo a biopsy procedure because of the high PPV (7%–8%) [15]–[16]. Dorrius MD [17] made a meta-analysis for the usefulness of breast MRI as a problem-solving modality in mammographic BI-RADS 3 lesions; 3 studies assessed the accuracy of MRI in mammographic BI-RADS 3 microcalcifications [4], [7], [15]; these studies reported an NPV of MRI between 76% and 97%; MRI can't be implemented as a diagnostic tool to evaluate mammographic microcalcifications. In our series, 21 BI-RADS category 3 microcalcifications were all benign, the NPV of mammography and MR were both 100% and the specificity of MR was lower than that of mammography, not significantly (100% VS 95.2%); MR did not show any improvement. A further study with larger number of cases is needed to evaluate whether MRI improves the diagnosis of category 3 microcalcifications.

#### Category 4 microcalcifications

BI-RADS category 4 (suspicious abnormality) includes lesions with high likelihood of malignancy: 2%–95%. Correct diagnosis of category 4 microcalcifications can minimize unnecessary biopsies. In our study, specificity, PPV and accuracy of MR was all significantly higher than that of mammography. Among 51 lesions of mammographic category 4, 25 lesions were degraded to BI-RADS 1∼3 proven of 23 benign and 2 malignant ones; that is to say, 82.1% (23/28) of benign microcalcifications could have been avoided biopsy after MR exam. Uematsu T [15] reported a PPV of 48% for mammography and 80% for MRI in patients with category 4 microcalcifications, which was in accordance with our results. Jiang Y [12] reported a PPV of 65.2% for mammography and 89.6% for MRI in patients with category 4 microcalcifications. Therefore MRI is an important imaging modality for assessing BI-RADS category 4 microcalcifications, adding significant value to mammography.

#### Category 5 microcalcifications

BI-RADS category 5 includes lesions with likelihood of malignancy over 95%. In this study, accuracy of mammography and MR were both 100%. MRI did not show advantage over mammography in the qualitative analysis of category 5 microcalcifications. Houserkova D [18] analyzed 35 lesions of mammographic category 5 with the sensitivity of MRI 94%, the accuracy 94%, PPV 100% and NPV 50%, and MRI didnot seem to be a prior choice to predict the presence of malignancy in patients with category 5 microcalcifications.

### Disadvantage of MRI

MRI provides tissue vascularity information that is unavailable from mammography. However, MR has several limitations and cannot replace biopsy. First, carcinoma without enhancement, especially DCIS, tend to be missed on MR [19]–[21]. two lesions with DCIS were missed in our study, one of which showed no enhancement on MRI, and the other mimic background parenchymal enhancement. The diagnosis of DCIS is still a crucial point that prevents us from clinical use of MRI in the diagnosis of microcalcifications [22]–[24]. Another issue is the correlation of the enhancement on MR with microcalcifications on mammography. Sometimes it is difficult to judge whether there is contrast uptake in the corresponding location on MRI. The position of the breasts is different on mammography where the breasts are compressed, while on MR the breasts are unforced. Lastly, interobserver variability in the practice of MR classification system is prominent [13].

### Study limitations

The fact that all calcifications that proved benign lacked follow-up was the limitation of our study. The great benefit of MRI is assessing tumor extent, multifocality and bilateral incidence of the carcinoma [18], which was not included in our study because only small percentage of tumors had pathologic size available in medical records, and that was another limitation of our study.

## Conclusions

Breast MRI has the potential to improve the diagnosis of category 4 microcalcifications on mammography with better specificity, PPV and accuracy. Among mammographic category 4 microcalcifications, about 82% of benign lesions can be degraded to BI-RADS 1∼3 by MRI. However for microcalcifications of category 3 and 5, MR exams donot show significant improvement compared to mammography. By performing additional breast MRI with mammography, the indications for biopsy or surgery may be altered.
